# Is ‘Self-Medication’ a Useful Term to Retrieve Related Publications in the Literature? A Systematic Exploration of Related Terms

**DOI:** 10.1371/journal.pone.0125093

**Published:** 2015-05-01

**Authors:** Ava Mansouri, Amir Sarayani, Asieh Ashouri, Mona Sherafatmand, Molouk Hadjibabaie, Kheirollah Gholami

**Affiliations:** 1 Research Center for Rational Use of Drugs, Tehran University of Medical Sciences, Tehran, Iran; 2 Department of Epidemiology and Biostatistics, School Of Public Health, Tehran University of Medical Sciences, Tehran, Iran; 3 Faculty of Pharmacy, Tehran University of Medical Sciences, Tehran, Iran; University of Illinois-Chicago, UNITED STATES

## Abstract

**Background:**

Self-Medication (SM), i.e. using medications to treat oneself, is a major concern for health researchers and policy makers. The terms “self medication” or “self-medication” (SM terms) have been used to explain various concepts while several terms have also been employed to define this practice. Hence, retrieving relevant publications would require exhaustive literature screening. So, we assessed the current situation of SM terms in the literature to improve the relevancy of search outcomes.

**Methods:**

In this Systematic exploration, SM terms were searched in the 6 following databases and publisher’s portals till April 2012: Web of Science, Scopus, PubMed, Google scholar, ScienceDirect, and Wiley. A simple search query was used to include only publications with SM terms. We used Relative-Risk (RR) to estimate the probability of SM terms use in related compared to unrelated publications. Sensitivity and specificity of SM terms as keywords in search query were also calculated. Relevant terms to SM practice were extracted and their Likelihood Ratio positive and negative (LR+/-) were calculated to assess their effect on the probability of search outcomes relevancy in addition to previous search queries. We also evaluated the content of unrelated publications. All mentioned steps were performed in title (TI) and title or abstract (TIAB) of publications.

**Results:**

1999 related and 1917 unrelated publications were found. SM terms RR was 4.5 in TI and 2.1 in TIAB. SM terms sensitivity and specificity respectively were 55.4% and 87.7% in TI and 84.0% and 59.5% in TIAB. “OTC” and “Over-The-Counter Medication”, with LR+ 16.78 and 16.30 respectively, provided the most conclusive increase in the probability of the relevancy of publications. The most common unrelated SM themes were self-medication hypothesis, drug abuse and Zoopharmacognosy.

**Conclusions:**

Due to relatively low specificity or sensitivity of SM terms, relevant terms should be employed in search queries and clear definitions of SM applications should be applied to improve the relevancy of publications.

## Introduction

Self-medication (SM) is a subcategory of self-care [1] and is a form of patients’ contribution to health decisions [2]. World Health Organization (1998) defined SM as “the selection and use of medicines by individuals to treat self-recognized illnesses or symptoms” [3]. SM is performed for about 80% of medical symptoms without professional supervision [3] and has become a common practice due to the rising of medical care and prescription drugs costs and patient empowerment [4]. Since the late 1980s, the rising trend of switching medicines status from prescription-only to over-the-counter has increased the use of non-prescription medicines for SM [5, 6]. Given that SM brings advantages and disadvantages for patients and health systems, it is now a major interest for researchers and policy makers [2].

The growing body of SM literature raises concerns about the best way of retrieving relevant publications in a reasonable amount of time. In one hand, excess of terms and phrases for the SM practice confronts us with complexity of understanding and working with the SM literature. Self-care, self-treatment, self-prescription, personal care, and drug self administration are among different terms which have been used in SM related publications [1, 7–16]. On the other hand, the rising amount of scientific publications, electronic journals and academic bibliographic databases have dramatically changed the way of distribution and sharing scientific information [17]. Although there have been some improvement in search engine algorithms, a common complaint still exists about the huge number of retrieved citations with low relevancy [17]. In order to achieve comprehensive search results while maximizing the odds of retrieving most relevant citations, it is recommended to create a list of possible synonyms and keywords related to the concept of the intended search [18]. Moreover, the sensitivity and specificity of the search terms are considered as important factors as revealed by a number of studies on the sensitivity and specificity of search filters to assess their methodological reliability [19].

There have been researches and reviews about terms and taxonomy in other concepts of health care, such as drug safety, medication errors and patient adherence to solve part of the misinterpretation and uncertainty resulted from terms overload and dissimilarity in their particular field as their primary intention [20–22]. However, SM has never been studied in this respect.

The purpose of this study was to analyze SM related and unrelated publications retrieved from well-known bibliographic databases. We also investigated terms associated with SM to help researchers improve their literature search relevancy.

## Methods

### Study Purpose and design

We carried out a systematic exploration of the relative usefulness of SM terms (self-medication and self medication) for retrieval of related publications. In overview we evaluate the pattern of publications related to SM. We investigate the characteristics of SM terms and SM synonyms and related terms in the literature over the study period.

The study was approved by the research council of Research Center for Rational Use of Drugs, Tehran University of Medical Sciences (TUMS).

### Data Sources and Search strategy

We performed a systematic search of the literature using several bibliography databases and publishers’ search engines including Web of Science, Scopus, PubMed, Google scholar, ScienceDirect (http://www.sciencedirect.com/), and Wiley online library (http://onlinelibrary.wiley.com/) without any limitation. The search strategy was “self medication” or “self-medication” (SM terms) within the title, abstract, keywords or MeSH according to each search engine characteristics. Search strategies for databases and publishers’ search engines are provided in the S1 Table. The search time span was up to April 2012.

### Screening protocol and inclusion/exclusion criteria

All citations were imported into an EndNote X4™ library. Duplicated records were removed by manual scanning by two reviewers in addition to matching the author, title, and publication year by software. Afterwards, two independent reviewers, AM and MS, screened the title and abstract to identify relevant publications. The reviewers judged the relevancy of citations based on WHO self-medication definition. Publications were excluded if they contained unrelated concepts; or did not contain English title and abstract. All types of publications regardless of study designs or article types were considered for inclusion in our study. A third reviewer, AA, judged the disagreements between the first two reviewers. Finally, we created two libraries of citations containing related or unrelated citations.

### Study objectives and analytical approaches

#### Objective 1: Trend of Publications

Before 1980, the number of publications per year was low and we focused on the yearly trend of publication since 1980. The simple proportion of related and unrelated publications was calculated for each year (i.e. Total SM related publication divided by total number of publications, and Total SM unrelated publication divided by total number of publications, respectively).

#### Objective 2: Evaluation of SM terms use in the literature

In this step we calculated the frequency of related publications which used SM terms (self medication or self-medication) in the title (TI). The proportion of related publications with the terms in TI to total number of related publications was calculated. The same procedure was carried out for the unrelated publications.

Relative risk (RR) was used to estimate the probability of SM terms use in the TI of related publications compared to the unrelated ones. In addition, we calculated the sensitivity and specificity of SM terms if they were used as keyword in ‘title search query’. All the aforementioned steps were carried out considering the SM terms in title or abstract (TIAB) of publications.

#### Objective 3: Evaluation of other terms relevant to SM practice

We looked for systematic and narrative reviews related to SM concepts in order to extract the terms relevant to SM practice which were used in their search strategies.

Afterwards, utilization of every extracted relevant term (RT) in TI of related and unrelated publications was investigated. Likelihood ratio positive (LR+) and likelihood ratio negative (LR-) were calculated for each RTs in TI of publications. LR indicates that how much the chance of article relevancy changes in the presence (LR+) or absence (LR-) of any RT. All the aforementioned steps were also carried out in TIAB of related and unrelated publications.

Based on LR concept, RTs with LR+>10 provide large and often conclusive increase in the likelihood of a publication relevancy. Terms with 5<LR+<10 point out a moderate increase in the probability and LR+<5 indicates a small effect on increasing the probability of publication relevancy.

#### Objective 4: Evaluation of authors’ assigned keywords

After classifying publications as related and unrelated, we searched for authors’ assigned keywords (KWs) of the related citations if not presented in the library. We used the ‘subject bibliography tool’ to extract the KWs bibliographic data. Afterwards, KWs were categorized based on city/country/continent, drug/pharmacologic group, disease/disorders, profession, herbs, field of science, organism, sex, and age group. We calculated the frequency of authors’ assigned KWs and the proportion of publications containing each KW among related publication.

#### Objective 5: Content evaluation of unrelated publications

Two reviewers categorized unrelated publications according to the study concept identified in the title and abstract.

## Results

**Fig 1:**
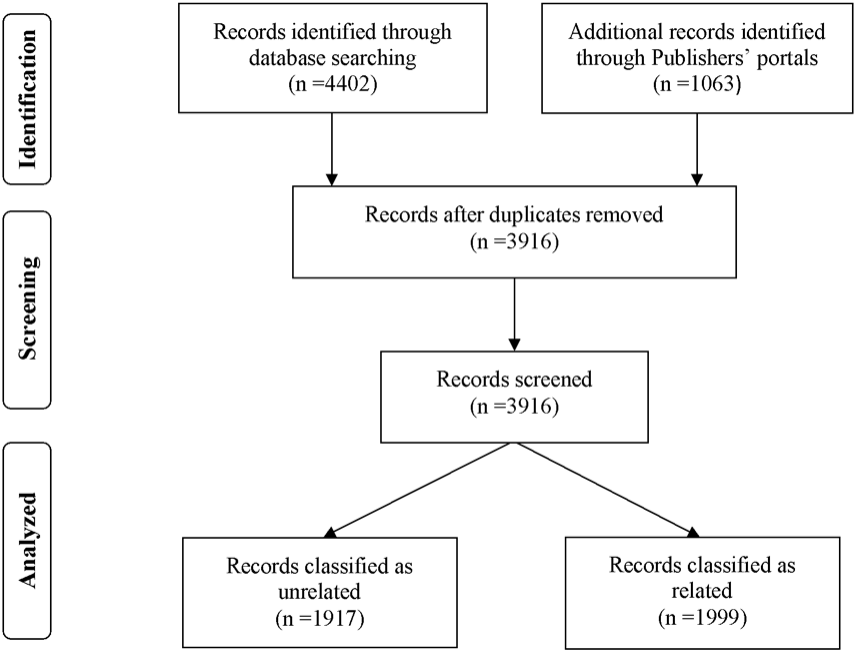
Self-medication flow diagram.

Initially, 5464 publications were identified. All duplicates were excluded (n = 1548), resulting in 3916 publications. Of these, 1917 were tagged as unrelated. Thereby, 1999 related publications remained for the final analysis after confirmation by three reviewers (Fig 1).

### Trend of Publications

**Fig 2:**
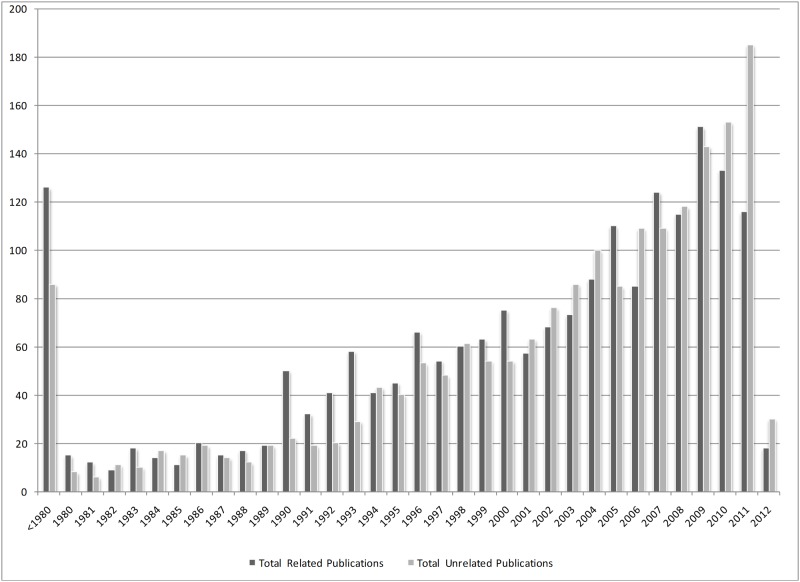
Total Related and Unrelated Publications in Year.

The first study was published in 1888. As it is shown in Fig 2, the number of total publications increased over the study period. The proportion of related to unrelated publications ranged between 0.63 and 2.27; while the number of unrelated publications increased since 1998, i.e. proportions < 1 in most years.

### Evaluation of “self-medication” terms use in the literature

Frequency of related and unrelated publications with SM terms in TI was 55.4% and 12.3% respectively (RR = 4.5). The RR indicates that the probability of using SM terms in the TI of related publications is 4.5 times to the unrelated publications. In TIAB, SM was used in 84.3% and 40.5% of related and unrelated publications respectively (RR = 2.1) (Table 1).

**Table 1:** Frequency of publications with SM terms in TI and TIAB of related and unrelated publications in each year.

Year	Total	Total	SM terms		Total	SM terms	
	publications	related publication	*TI*	*TIAB*	unrelated publication	*TI*	*TIAB*
<1980	212	126	113	118	86	30	36
1980	23	15	13	15	8	0	0
1981	18	12	10	12	6	0	0
1982	20	9	8	9	11	1	1
1983	28	18	15	16	10	2	3
1984	31	14	11	14	17	2	5
1985	26	11	11	11	15	4	5
1986	39	20	18	18	19	4	4
1987	29	15	13	14	14	4	6
1988	29	17	13	14	12	3	5
1989	38	19	14	16	19	2	4
1990	72	50	44	45	22	3	5
1991	51	32	22	28	19	3	7
1992	61	41	28	34	20	3	5
1993	87	58	30	50	29	5	15
1994	84	41	28	37	43	7	16
1995	85	45	31	45	40	5	24
1996	119	66	33	54	53	5	25
1997	102	54	33	45	48	5	16
1998	121	60	33	49	61	9	24
1999	117	63	26	50	54	3	28
2000	129	75	34	63	54	2	19
2001	120	57	26	40	63	6	32
2002	144	68	25	49	76	9	30
2003	159	73	30	59	86	10	42
2004	188	88	31	58	100	6	35
2005	195	110	51	85	85	8	34
2006	194	85	34	63	109	9	47
2007	233	124	62	98	109	12	45
2008	233	115	45	100	118	12	42
2009	294	151	84	135	143	29	68
2010	286	133	60	112	153	8	52
2011	301	116	68	108	185	18	79
2012	48	18	11	14	30	6	17

**Fig 3:**
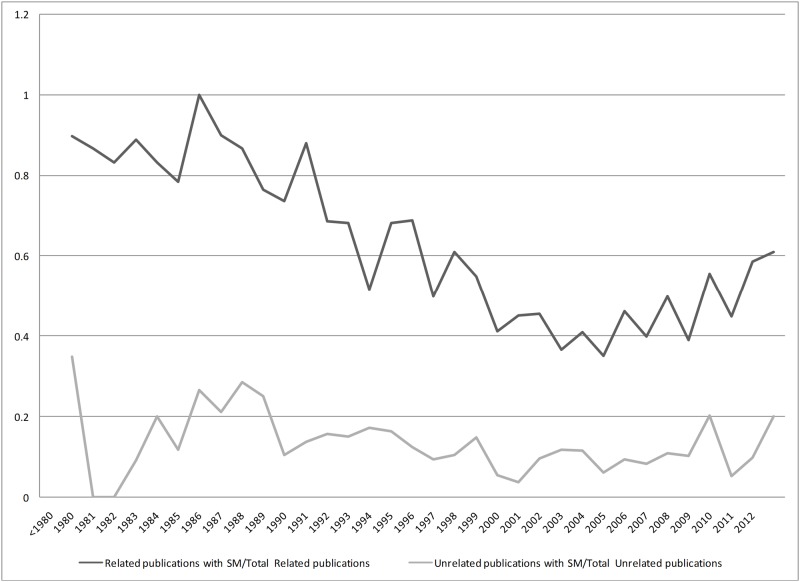
Proportion of Related and Unrelated Publications Which Used SM Terms in Their TI.

**Fig 4:**
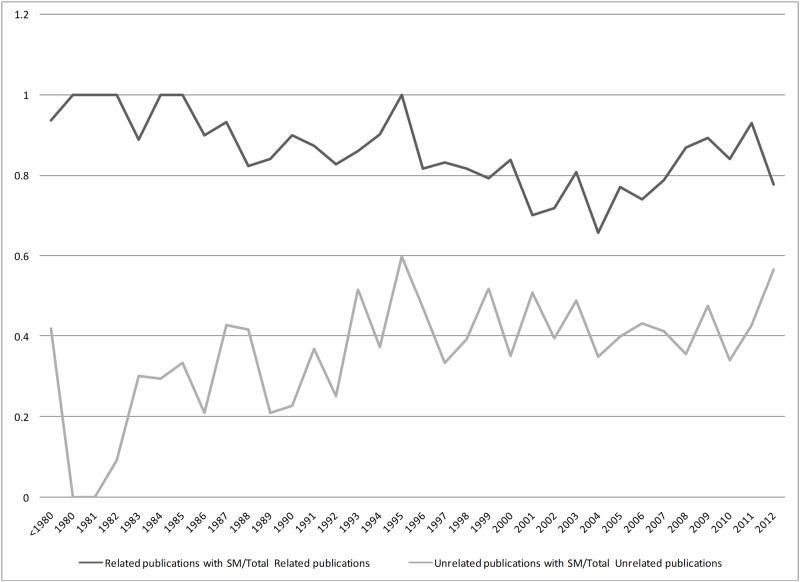
Proportion of Related and Unrelated Publications Which Used SM Terms in Their TIAB.

The proportion of related and unrelated publications which used SM terms in their TI or TIAB is illustrated across time in Figs 3 and 4. Among related publications, use of SM in TIAB has a similar trend to SM in TI with less variation (0.35–1 in TI vs 0.66–1 in TIAB). Presence of SM terms in the TIAB of unrelated publications had an increasing trend until 1995 but it became almost constant after 1998.

#### Sensitivity and specificity

We estimated that SM in TI would have a sensitivity of 55.4%. This suggests that if search strategy includes word SM in TI, about 55% of total related publications would be retrieved. Moreover, the specificity of SM in TI would be 87.7%; this means that approximately 12% of retrieved publications would be unrelated.

A hypothetical search query of SM in TIAB would have a sensitivity and specificity of 84.0% and 59.5% respectively.

### Evaluation of terms relevant to self-medication practice

Nineteen RTs were identified from the search strategies of ten narrative and systematic reviews (theses reviews and extracted RTs are listed in S2 Table). We found sixteen of these RTs were to be used in the SM related publications. The LR+ and LR- of each RT are provided in Table 2.

**Table 2:** LR+ and LR- of relevant terms in TI and TIAB of related and unrelated publications.

Relevant terms	TI				TIAB			
	Related*	Unrelated**	LR +	LR -	Related	Unrelated	LR +	LR -
OTC	35	2	16.78	0.98	182	12	14.54	0.91
Over-The-Counter Medication	17	1	16.30	0.99	32	3	10.23	0.99
Over-The-Counter	97	7	13.29	0.95	279	38	7.04	0.88
Nonprescription	28	2	13.43	0.99	102	19	5.15	0.96
Self-Care	31	4	7.43	0.99	86	21	3.93	0.97
Self-Treatment	27	8	3.24	0.99	85	19	4.29	0.97
Self-Prescription	5	2	2.40	1.00	13	3	4.16	1.00
Self-Medicating	4	2	1.92	1.00	30	20	1.44	1.00
Over-The-Counter Drug	17	0		0.99	39	5	7.48	0.98
Nonprescription Drug	11	0		0.99	32	8	3.84	0.99
Over-The-Counter Medicine	7	0		1.00	20	2	9.59	0.99
Nonprescription Medication	10	0		0.99	18	1	17.26	0.99
Over-The-Counter Preparation	2	0		1.00	3	2	1.44	1.00
Self-Efficacy	2	0		1.00	11	25	0.42	1.01
Self-Administration	11	21	0.50	1.01	26	47	0.53	1.01
Drug Self-Administration	1	2	0.48	1.00	1	6	0.16	1.00

* Total related publications = 1999.

**Total unrelated publications = 1917.

RTs such as “OTC” and “Over-The-Counter Medication”, which had the highest LR+ in the TI of publications, if added to the search strategy, will provide the most conclusive increase in the probability of publications relevancy. This is also applicable to the terms such as “Nonprescription Medication” and “OTC” in TIAB. RTs with LR+ of 0.5–1, e.g. “Self-Administration” and “Drug Self-Administration”, have no or the minimal change in likelihood of publication relevancy.

LR- of all the RTs is close to 1 (0.95–1.01) which demonstrates that the absence of RTs in the search strategy would have no considerable effect in the likelihood of findings relevancy. This means that if there is a publication in our findings which RTs are not used in its TI or AB, it cannot be decided that the publication is probably related or unrelated.

### Evaluation of authors’ assigned keywords

Manual searches for publications with KWs yielded to 825 articles with a total of 2251 KWs. The ‘frequent KWs’, i.e. used in more than 10 publications, are listed in the Table 3. We also separately count the KWs in categorized groups and the most frequent KWs are as follow. In age groups, elderly (n = 44, 5.33%), adult (n = 41, 4.96%) and pediatrics (n = 28, 3.93%) were frequently cited. In pharmacologic category, antibiotics (n = 122, 14.78%), analgesics (n = 93, 11.27%) and supplements (n = 18, 2.18%) were the most frequent KWs. In disease/disorder category, majority of KWs were categorized under Infectious disease (n = 86, 10.42%), different types of headache (n = 55, 6.66%), psychiatric (n = 52, 6.3%) and GI disorders (n = 39, 4.72%).

**Table 3:** Number of the articles with frequent KWs.

keywords	N	(%)*	keywords	N	(%)	keywords	N	(%)
Self-medication	365	44.24	Pharmacist	34	4.12	Safety	23	2.78
Antibiotic	82	9.93	Pain	34	4.12	Drug interaction	22	2.66
Over-the-counter	69	8.36	Analgesics	31	3.75	Pharmacoepidemiology	20	2.42
Pharmacy	65	7.87	Student	30	3.63	Compliance	20	2.42
Nonprescription	59	7.15	Drug use	29	3.51	Alternative medicine	19	2.30
Prescription	53	6.42	Elderly	26	3.15	Children	17	2.06
Malaria	47	5.69	Epidemiology	26	3.15	Pregnancy	16	1.93
Herbs	47	5.69	Migraine	26	3.15	Depress	15	1.81
Self care	40	4.84	Prevalence	23	2.78	Self-treatment	14	1.69
Survey	40	4.84	Drug utilization	23	2.78	Drug therapy	10	1.21
Headache	35	4.24	Adolescent	23	2.78			
OTC	34	4.12	Knowledge	23	2.78			

*(% = N/ 825).

### Content evaluation of unrelated publications

After categorizing unrelated SM publications, they could be classified as self-medication hypothesis (SMH), addiction and alcohol consumption, drug abuse and case reports of toxicity due to overdoses or non-medical products use, and animal self-medication (Zoopharmacognosy).

## Discussion

In the present study, we investigated the trend of SM related and unrelated publications retrieved by a simple search query. We also calculated the sensitivity and specificity of the query to understand the extent to which the SM terms have been used to describe concepts other than ‘self-medication’ as defined by the WHO. In addition, we investigated a group of RTs to find possible strategies to improve relevancy of search results.

Titles and abstracts are important parts of publications because they are commonly indexed in bibliographic databases [23] and researchers use them to check publications relevancy during literature search [24, 25]. In addition, terms used in the title or abstract of publications should be reflective of the study concept, i.e. SM, to increase the visibility of citations [25, 26]. Therefore, we focused our analysis of relevancy, sensitivity, and specificity on titles and abstracts of the retrieved citations.

The numbers of related and unrelated publications increased over time. During 90s, related publications comprised the majority of citations and the WHO definition of SM was introduced in 1998. We did not observe a major effect of the WHO guidance on the proportion of related to unrelated publications during 2000s. However, a positive shift in the trend of SM terms utilization in the related publications could be observed since early 2000s. The trend of SM terms use in the unrelated publications did not change significantly over time.

As it was expected, the probability of using SM terms in TI or TIAB of related publications is relatively higher than the unrelated ones over the study period. Nevertheless, the sensitivity of SM terms in TI was relatively low (approximately 50%) which indicates that half of the related publications had not included the SM terms in TIs. However, a relatively high specificity of 88% showed that publications with SM terms in TI will probably be related to SM. On the other hand, SM terms in TIAB would result in 84% sensitivity and 60% specificity.

It has been stated that by searching simple queries (e.g., single term) thousands of documents would be retrieved and ranking their relevancy is a huge problem [17]. We encountered with the same set of problems in the present study.

We aimed to assess the effect of using RTs in addition to the simple search query of SM terms as one of the strategies for improving the relevancy of search outcomes. So in order to collect an inclusive list of synonyms and keywords as suggested [18], we used narrative and systematic reviews to extract the terms relevant to SM.

If we use one RT in a search strategy, the existence of the term in a group of citations could mean that they are more likely to be relevant in comparison to those without the term. Based on the LR concept, as the LR+ increases, the probability of relevancy for the publication with the RT rises. Nevertheless, LR- figures close to 1 for the RTs not appearing in the publication may not imply of the probability of the publication being related or unrelated.

We observed changes in LR+ for some RTs when they are placed in TI compared to TIABs. This suggests a possible change in their effect on the probability of publication relevancy. For instance, when “Nonprescription” is in TI instead of TIAB this would increase the likelihood of being related from moderate to large (TIAB’s LR+ = 5.15, TI’s LR+ = 13.43). In other words if it is searched in TI, the probability of the publications with the RT in their title being related to SM is higher and more conclusive.

As we learned in this study, each RT with its sensitivity and LR gives us a part of related publications; some of the related publications are overlapped (several RTs were used in one publication), and also there are many relevant publications which did not use any RT in addition to the SM terms. So these terms on their own or in addition to other terms could not provide all of the related publications. This would lead researchers to use multiple RTs at a time in search strategy to increase the relevancy of results.

Keywords are terms (words or concise-phrase) which expose the main focus of the study [27] and we used the authors' assigned KWs to categorizes studies. Elderlies were the most common studied population according to KWs. The researchers' interest in this age category could be justifed by the higher prevalence of SM practice and particularly the vulnerability to adverse eventsamong elderlies [28, 29]. Antibiotics and infectious diseases were frequently studied. Since emergence of antibiotic resistance is a global public health concern, it has drawn attention towards SM as an important underlying risk factor [30, 31]. Regarding therapeutic categories, analgesics were commonly studied considering several studies have reported that patients self medicate with analgesics quite frequently [3, 28, 29, 32]. Finally, headache was a common health condition of SM studies and this is also consistent with patients' SM practice studies [3, 28, 33].

A content evaluation of the unrelated library showed that the most repetitive unrelated concepts were about alcohol consumption and addiction (self-medication hypothesis or SMH) [34, 35], drug abuse, case reports of toxicity due to overdoses or non-medical products use [36], and animal Self-medication (zoophamacognosy) [37]. Although these concepts would be easily found in the routine search for SM and they have mostly used SM terms in their manuscripts to demonstrate their concept, but they embrace a range of unrelated definitions, hence they are considered as disturbing contexts.

## Limitations

Our search was limited to English language only and our findings should not be extrapolated to all published literature. However, English publications are the mainstay of bibliography searches. We judged the relevancy of publications only by screening titles and abstracts because they are used by bibliographic databases to cite publications. In addition, this approach is accepted for rigorous systematic reviews for early screening of search outcomes. The study purpose was to assess the characteristics of “self-medication” and “self medication” terms in related publications. Since there is no specific database which includes all the related SM publications we were obligated to use the most common terms in this concept (SM terms) to make our own database for further evaluation.

## Conclusion

Use of SM terms alone cannot provide researchers with desirable search outcomes due to relatively low specificity and or sensitivity. Researchers should employ other possible RTs to improve publications relevancy. Due to overlap in the utilization of SM terms (self-medication and self medication) in different concepts with specific different definitions such as zoophamacognosy and SMH, searching SM terms leads to deterioration in the relevancy of search results. This is happening despite the fact that self-medication has been defined by WHO. So there is a need for clearer definitions and limited utilization of different SM applications.

## Supporting Information

S1 Table Search strategies for evaluated databases and publishers’ search engines. (PDF)

S2 Table Extracted SM related terms of systematic and narrative reviews about self-medication. (PDF)
