# Supplementary material for: Is ‘Self-Medication’ a Useful Term to Retrieve Related Publications in the Literature? A Systematic Exploration of Related Terms
Source: PLoS One. 2015 May 1;10(5):e0125093. doi: 10.1371/journal.pone.0125093 (PMC4416799; doi:10.1371/journal.pone.0125093)
Supplement: S1 Table — (PDF) [file pone.0125093.s001.pdf]

S1 Table. Search strategies for evaluated databases and publishers’ search engines

Database	Search Strategy
PubMed	((self medication[Title/Abstract] OR "self-medication"[MeSH Terms]) OR self-medication[Title/Abstract]) OR "self-medication"[MeSH Terms]
Scopus	TITLE-ABS-KEY("self-medication") OR TITLE-ABS-KEY("self medication")
ScienceDirect	TITLE-ABSTR-KEY("self-medication") or TITLE-ABSTR-KEY("self medication")
Wiley online library	"self medication" in Article Titles OR "self medication" in Abstract OR "self medication" in Keywords OR "self-medication" in Article Titles OR "self-medication" in Abstract OR "self-medication" in Keywords
Web of Science	TOPIC: ("self-medication") OR TITLE: ("self-medication") OR TOPIC: ("self medication") OR TITLE: ("self medication")<br>Timespan: All years.<br>Search language=Auto
Google scholar	allintitle: "self medication" OR "self medication"
