# Supplementary material for: Is ‘Self-Medication’ a Useful Term to Retrieve Related Publications in the Literature? A Systematic Exploration of Related Terms
Source: PLoS One. 2015 May 1;10(5):e0125093. doi: 10.1371/journal.pone.0125093 (PMC4416799; doi:10.1371/journal.pone.0125093)
Supplement: S2 Table — (PDF) [file pone.0125093.s002.pdf]

S2 Table. Extracted SM related terms of systematic and narrative reviews about self-medication

**Systematic/ Narrative review**	**Extracted related terms**
Godfrey et al. [1]	Self-Care<br>Self-Care Skills<br>Self-Administration<br>Self-medication<br>Self-Treatment<br>Self efficacy<br>Personal Care
Kaizer et al. [7]	Self-medication<br>Self-Administration
Ryan et al. [8]	NA
Elliott et al. [9]	Self-Administration
McKenna et al. [10]	Over-The-Counter Medication<br>Nonprescription Medication<br>Over-The-Counter Preparation
Schroeder et al. [11]	Drugs, Nonprescription<br>Self medication<br>Over-The-Counter<br>OTC<br>Nonprescription
Montgomery et al. [12]	Self-medication<br>Self-Treatment<br>Self-Prescription
Brata et al. [13]	Self-medication<br>Self-Treatment<br>Self-Care<br>Over-The-Counter Medic*<br>Nonprescription Drug*
Wimpenny et al. [14]	Self-Medicating<br>Self-medication<br>Drug Self-Administration<br>Medicine Self-Administration<br>Self-Administration
Dedy et al. [15]	Self-Care<br>Self-medication<br>Over-The-Counter Medicine<br>Nonprescription Medicine
Morgan et al. [16]	Over-The-Counter<br>OTC<br>Nonprescription<br>Self-medication
